# Accurate prediction of inter-protein residue–residue contacts for homo-oligomeric protein complexes

**DOI:** 10.1093/bib/bbab038

**Published:** 2021-03-05

**Authors:** Yumeng Yan, Sheng-You Huang

**Affiliations:** School of Physics, Huazhong University of Science and Technology, Wuhan, Hubei 430074, PR China; School of Physics, Huazhong University of Science and Technology, Wuhan, Hubei 430074, PR China

**Keywords:** protein–protein interaction, homo-oligomers, protein–protein docking, inter-protein contact prediction, deep learning

## Abstract

Protein–protein interactions play a fundamental role in all cellular processes. Therefore, determining the structure of protein–protein complexes is crucial to understand their molecular mechanisms and develop drugs targeting the protein–protein interactions. Recently, deep learning has led to a breakthrough in intra-protein contact prediction, achieving an unusual high accuracy in recent Critical Assessment of protein Structure Prediction (CASP) structure prediction challenges. However, due to the limited number of known homologous protein–protein interactions and the challenge to generate joint multiple sequence alignments of two interacting proteins, the advances in inter-protein contact prediction remain limited. Here, we have proposed a deep learning model to predict inter-protein residue–residue contacts across homo-oligomeric protein interfaces, named as DeepHomo. Unlike previous deep learning approaches, we integrated intra-protein distance map and inter-protein docking pattern, in addition to evolutionary coupling, sequence conservation, and physico-chemical information of monomers. DeepHomo was extensively tested on both experimentally determined structures and realistic CASP-Critical Assessment of Predicted Interaction (CAPRI) targets. It was shown that DeepHomo achieved a high precision of >60% for the top predicted contact and outperformed state-of-the-art direct-coupling analysis and machine learning-based approaches. Integrating predicted inter-chain contacts into protein–protein docking significantly improved the docking accuracy on the benchmark dataset of realistic homo-dimeric targets from CASP-CAPRI experiments. DeepHomo is available at http://huanglab.phys.hust.edu.cn/DeepHomo/

## Introduction

As one of the most important biological macromolecules in living organisms, proteins have evolved to conduct diverse cellular functions, from serving as reaction catalysts to regulating DNA translation and transcription. Proteins rarely work in monomeric form but are rather assembled in homo-oligomers or hetero-oligomers to perform their biological functions [1, 2]. The structural characterization of protein–protein interactions and high-order assemblies is therefore crucial to elucidate the molecular mechanisms behind them and understand the related biological processes [3, 4]. Despite the great progress in experimental determination of macromolecular structures [5–7], the number of protein structures that have been experimentally determined and deposited in the Protein Data Bank (PDB) [8] is still limited. Compared with the limited structures in the PDB, the genetic sequential information has increased dramatically as the result of high-throughput sequencing technologies and large-scale genome projects [9, 10]. Taking advantage of the huge sequence information through sophisticated statistical models provides a valuable alternative to determine the structures [11–14]. Recently, direct-coupling analysis (DCA) [15–19] and similar tools [20, 21], which try to distinguish direct from indirect correlation effects, have been developed to improve the performance of residue–residue contact prediction from multiple sequence alignments (MSAs). Such computational methods have proven to be very useful in monomer protein structure prediction in the Critical Assessment of protein Structure Prediction (CASP) experiments [22–25].

Subsequently, such DCA methods have been extended from the contact prediction within the monomer protein structure to residue–residue contact prediction at the hetero protein–protein interface [26, 27]. Despite some successes, there is still a challenge for such coevolution-based contact prediction in protein–protein interactions. That is, how to create a joint MSA of high quality, in which each line contains the sequences of a pair of interacting proteins [28]. Constructing an MSA for individual proteins is relatively easy using protein sequence searching software like HHblits [29] or PSI-blast [30]. However, generation of the joint MSA from the two MSAs of interacting proteins is difficult due to the existence of paralogs [28, 31–33]. In bacteria, the two individual MSAs can be concatenated according to their genomic distance because the genes coding for interacting proteins are often colocalized in the same operon [26, 27]. For eukaryotes, phylogeny tree may be used to concatenate MSAs [34]. Recently, two iterative methods have been presented to identify specific interacting paralogs by maximizing the DCA signals [31, 32]. However, all above methods have limitations because the produced MSA does not have sufficiently divergent sequences or contains many incorrect paired sequences. Besides the DCA models, some machine/deep learning methods have been developed to predict the residue–residue contacts between protein–protein interfaces with [34] or without producing the joint MSA for the complex [35–38]. However, the accuracy of these methods is still relatively low.

To avoid the joint MSA challenge, Uguzzoni *et al.* have focused on the interactions between homo-oligomers rather than hetero-oligomers, for which the produced individual MSA was directly used to perform the statistical analysis on the basis of the assumption that the protein in each line of the MSA also forms homo-oligomeric interface like the queried protein [39]. This avoids the challenge of creating of the joint MSA for residue–residue contact prediction in hetero-oligomeric protein interface. However, another problem presents, that is how to distinguish the intra-protein and inter-protein contacts in a homo-oligomer [14]. Uguzzoni *et al.* assumed that the predicted residue pair contacts with long intra-monomer distances are inter-protein contacts. Recently, a similar method has been proposed by the Cheng’s group to predict the inter-protein contacts of homo-oligomers [40]. The true intra-protein contacts obtained from the monomer structure and their neighbors within a square region of a certain size were removed from the contact maps predicted by the intra-protein contact predictor DNCON2 [41], and the retained contacts were considered as the predicted inter-protein contacts. However, using the monomer structure to filter all the intra-protein contacts may neglect the residue–residue contacts that are both intra-protein and inter-protein.

Recently, deep learning has achieved great successes in intra-protein residue–residue contact prediction and demonstrated high accuracy in the 12th and 13th Critical Assessment of protein Structure Prediction (CASP12 and CASP13) challenges [42–49]. However, there are significant differences between monomer proteins and homo-oligomeric complexes in terms of contact prediction [39]. First, for homo-oligomeric interface contact prediction, it is difficult to remove the potential intra-protein contact noises that exist in the MSA. Second, the number of inter-protein contacts is far smaller than that of intra-protein contacts for homo-oligomers. To overcome these challenges, we have here presented a deep learning model to predict residue–residue contacts across homo-oligomeric interfaces, named as DeepHomo, by integrating evolutionary coupling, sequence conservation, structural features, and physico-chemical information of monomers. With the help of the integrated structural features and the deep learning network, DeepHomo can robustly distinguish the inter-protein contacts from the intra-protein contact noises in the MSA features and achieve a better performance than traditional DCA and machine learning (ML)-based methods. DeepHomo was extensively tested on 300 diverse homo-oligomeric proteins from the PDB and 28 realistic targets from recent CASP-Critical Assessment of Predicted Interactions (CAPRI) challenges [50–52].

## Materials and Methods

### Deep learning architecture

**Fig. 1:**
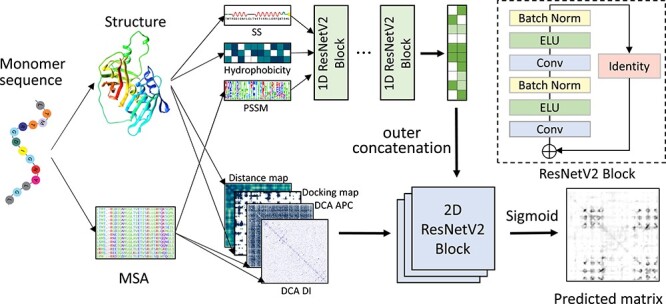
The framework of the DeepHomo model. The model consists of two components: a 1D convolutional neural network with 1D features as input, which contain secondary structure (SS), hydrophobicity features and PSSM matrix (top), and a 2D convolutional neural network (bottom) with 2D features as input, which include distance map, docking map and DCA scores.

Figure 1 shows the workflow of our deep learning-based inter-protein contact prediction for homo-oligomeric complexes (DeepHomo). DeepHomo starts with a monomer structure that may be taken from an experimental structure or predicted by a third-party protein structure prediction method like I-TASSER [53, 54]. As shown in Figure 1, 1D convolutional neural network is first used to capture the sequential context and extract the high-dimensional features from sequential features. Then the extracted high-dimensional sequential features are converted into pairwise features through outer concatenation as used in the RaptorX-contact study [42]. The converted features are concatenated with input pairwise features together. At last, the 2D convolutional neural network is used to capture pairwise context and output the predicted contact probability for each residue pair.

Like other deep learning methods proposed for monomer structure contact prediction, the central part of our convolutional neural network (CNN) is a stack of residual network (ResNet) blocks [55, 56]. ResNet has been widely used in computer vision and protein contact prediction because the shortcut connection added in ResNet makes the training of extremely deep CNN possible. Different with RaptorX-contact, we used ResNet v2 [56] as the basic network block for both 1D and 2D networks in this study, which has been shown to make training easier and improve generalization. The ResNet v2 block consists of two batch normalization layers, two activation layers, two convolutional layers and a shortcut connection between the input and output of the last convolutional layer. If the input tensor of the block has a different dimension with the output one of the convolutional layer, a convolutional layer with kernel size of $1 \times 1$ will be used to change the dimension of the input tensor, that is the identity layer as shown in Figure 1.

The 1D CNN contains six 1D ResNet v2 blocks with an increased number of filters. A convolutional layer with two $1 \times 1$ filters is used to compress the output tensor of the 1D network. The kernel size of the 1D network is set to 17. The numbers of filters for the six blocks in the 1D network are set to 35, 40, 45, 50, 55 and 60, respectively. The 2D CNN is stacked by 36 2D ResNet v2 blocks with nine groups of filters. The numbers of filters for the nine groups of 2D blocks are set to 32, 32, 48, 64, 64, 64, 48, 32 and 32, respectively. The output block consists of a batch normalization layer, an activation layer and a convolutional layer with $1 \times 1$ kernel where a sigmoid activation is used to convert the predicted probability in the range 0 to 1. The convolutional kernel size of the 2D network is set as $3 \times 3$. The exponential linear unit is utilized as the activation function in the network. All the convolutional operations are with zero padding to maintain the size of the predicted contact map. As the training parameters in the network are independent to the size of input, our model can take variable-length proteins as input.

### Data sets

Protein homo-oligomers have different symmetry types and stoichiometry, which may form different residue contacts at the interfaces. To avoid the effect of multiple interfaces and reduce potential noise, we focused on the homo-dimeric proteins with C2 symmetry type, which is the largest class of the homo-oligomers [2]. First, we queried all the biological assemblies in the PDB with the following criteria: (i) they are assigned C2 symmetry by the authors; (ii) the resolution is better than 3.0 Å; (iii) the biological unit only contains two protein chains; (iv) the lengths of protein chains range from 50 to 500. All the queried biological assemblies were checked using blast [57] to make sure that the two chains in one assembly share more than 99% sequence identity. Next, all the candidate assemblies were clustered using MMseqs2 [58, 59] with a sequence identity cutoff of 30% to remove the redundancy. The assembly with the best resolution was chosen as the representative of the cluster. Then, the interface area was calculated for each assembly structure and only the structures with interface more than 1000 Å$^{2}$ were retained. The interface area was measured as half of the change in the solvent accessible surface area of two monomers upon binding ($\Delta $SASA), where $\Delta $SASA was calculated by the sum of SASA of the two monomers minus the SASA of the dimer. Finally, the candidates with the sequence identity of more than 30% with any target in the CASP-CAPRI test set were also removed to guarantee that there was no homology between the CASP-CAPRI test set and the training set. This yielded a final data set of 4132 homo-dimeric complex structures, of which 3532 structures were used as the training set, 300 structures as the valid set and 300 structures as the test set.

In addition, another independent data set, called CASP-CAPRI test set, was also constructed to evaluate the performance of our deep-learning model in realistic applications. The joint CASP-CAPRI challenge has been established since 2014, which is a double blind experiment and aims to assess the computational methods of modelling protein assemblies in the community [50–52]. We collected all the homo-dimeric targets from recent four CASP-CAPRI challenges that have experimental complex structures available, yielding a total of 27 targets. In addition, we also split the CAPRI target T149 (CASP target T0999) into two interacting targets T149_D1 and T149_D4 according to the domain definition in the CASP experiment due to the large size of 1589 residues. Finally, the CASP-CAPRI test set consists of 28 homo-dimeric targets with known experimental structures. To test the realistic performance of our DeepHomo model, for each target in CASP-CAPRI test set, the first Zhang-Server model, i.e. Zhang-Server-TS1, in the CASP experiments was used as the input monomer structure of DeepHomo during the evaluations. The quality of the monomer model is measured by its TM-score [60–62].

### Input features

Similar to the inter-protein contact definition in the previous studies [26, 27, 39], two residues from different monomers are considered as in contact if any two heavy atoms from the two residues are within 8 Å. Another distance threshold of 6 Å as adopted by BIPSPI [38] was also used to train our model and the results were shown in the Supplementary Material. Multiple features were used in our deep learning model to predict the residue contacts. These features can be grouped into two categories. One is one-dimensional (1D) sequential features including protein sequence profile like position-specific scoring matrix (PSSM), 8-state secondary structure types and three hydrophobicity scales of each amino acid. The other is two-dimensional (2D) pairwise features, including residue–residue distance map of monomer structure, docking map created by our FFT-based docking program and direct co-evolutionary information calculated by CCMpred [18].

Give a monomer of $L$ amino acids, we first built an MSA for its sequence by running HHblits [29] with a minimum coverage of 40% and a maximum pairwise sequence identity of 99% with the query sequence against the UniRef30_2020_03 database [63] by three iterations. The e-value threshold was set as the default one of 0.001. Then, according to the constructed MSA, the raw and average product correction (APC)-corrected direct coupling scores were calculated by running CCMpred. The protein sequence profile (PSSM) was also generated. The direct co-evolutionary feature was represented as a matrix with dimension $L \times L \times 2$ and the PSSM feature was represented as a matrix with size of $L \times 20$.

Besides the sequence for each target, the 3D monomer structure was also used to produce the input features. It is expected that the features derived from monomer structures are helpful to distinguish inter-protein from intra-protein contacts. The Dictionary of Protein Secondary Structure (DSSP) program [64] was first used to assign the secondary structure type for each amino acid of the monomer structure. Then, the assigned secondary structure types were converted into a matrix with dimension $L \times 8$ according to one-hot encoding. The ‘ACC’ column in the output of DSSP, which represents the water molecules in contact with this residue, was also used as a hydrophobicity feature. The absolute solvent accessible surface area of each residue calculated by the Naccess program [65] was used as another hydrophobicity feature. DSSP uses Shrake and Rupley’s method [66] to calculate the solvent accessible surface area where the surface is approximated by a set of points, while Naccess adopts Lee and Richards’s method [67] where the surface is approximated by the outline of a set of slices [68]. To better describe the hydrophobicity feature, we have used the both features in our model. The Wimley–White whole residue hydrophobicity scale [69] was used as the third hydrophobicity feature. The pairwise distance map of the monomer structure consisted of the minimal heavy atom distances of all residue pairs with size of $L \times L \times 1$. At last, the docking map was generated from a modified version of our in-house FFT-based docking program, HSYMDOCK [70, 71]. The angle interval was set as 6$^{\circ }$, which resulted in 960 evenly distributed rotations in Euler space. For each rotation, the top 100 translations according to the shape complementary score were used to generate the docking map. Specifically, for each binding mode, i.e. one predicted complex structure, a contact map for the interaction of two chains was constructed with the same definition as described above. Then, the union of all the 96 000 contact maps was taken as the docking map with size of $L \times L \times 1$. For each target in the training, valid and test set, the monomer structure was directly exacted from the experimental complex structure. For each target in the CASP-CAPRI test set, the top Zhang-Server model from CASP was used as the monomer structure.

### Implementation

The Keras with the Tensorflow as backend was used to implement our deep learning model. The hyper-parameters were set as follows: mini-batch size: 1, optimizer: Adam [72], learning rate: 0.001, and L$_{2}$-norm regularization coefficient: 1e-4. The L$_{2}$-norm regularization was applied by adding a weight decay to each convolutional layer. The learning rate decay strategy was set as follows. If the loss of the valid set stops improving in two epochs, the learning rate will reduce by a factor of 0.2 and the minimal learning rate was set as 1e-6. Due to the extremely unbalance between non-contact residue pairs and the contact ones, here we used the Focal Loss [73] as the loss function to train our deep learning model. The loss function for each residue pair of $i$ and $j$ is described as follows.

(1) \begin{align*}& \mathrm{FL}\left(p_{\mathrm{t}}\right)=-\alpha_{\mathrm{t}}\left(1-p_{\mathrm{t}}\right)^{\gamma} \log \left(p_{\mathrm{t}}\right) \end{align*}

where $\alpha _{t}$ and $p_{t}$ are defined as

(2) \begin{align*}& \alpha_{\mathrm{t}}=\left\{\begin{array}{ll} \alpha & y=1 \\ 1-\alpha & y=0 \end{array}\right. \end{align*}

and

(3) \begin{align*}& p_{\mathrm{t}}=\left\{\begin{array}{ll} p_{\mathrm{ij}} & y=1 \\ 1-p_{\mathrm{ij}} & y=0 \end{array}\right. .\end{align*}

Here, $p_{ij}$ is the predicted contact score of residue $i$ and residue $j$, $\alpha $ is the parameter to balance the importance of positive and negative examples, and $\gamma $ is the tunable focusing parameter to focus learning on hard examples and down-weight the numerous easy examples. In our model, $\alpha $ was set as 0.25 and $\gamma $ was set to 1.5, Due to the limited graphics processing unit (GPU) memory, the maximum length of the protein fed into the network was limited to 400. If the length is more than 400, a continuous subsequence with length of 400 is randomly sampled to represent the protein. The model was trained on one K80 GPU and after around 20 epochs (about 24h), the model converged to a stable solution.

As homo-dimeric protein often has a C2 symmetry type, the ideal contact map for a homo-dimeric interface is diagonally symmetric. Hence, the labels and the input features used to train our model have to be operated to ensure the symmetry. For the input pairwise features, the transposed and untransposed matrixes were averaged to get the final input symmetric matrix. For the labels of the contact map, if the residue pair ($i,j$) was with label of 1, the residue pair ($j,i$) would be set to 1 too, and vice versa. Mask records were created for missing residue pairs in the complex structure to ignore those missing residue pairs in the loss function.

### Evaluation criteria

The performance of residue–residue contact prediction was measured by three parameters. One is the precision, which is defined as the percentage of true positive (TP) contacts among the top $n$ predicted contacts. As a few correct restraints may significantly help filter the correct binding modes for molecular docking [74], so the precision of the top contact predictions is crucial for complex structure prediction. The average precision of all the targets was used to represent the performance of a model on the data set. Besides the precision of the top $N$ contact predictions, we also evaluated the top $L/K (K=30, 20, 10, 5, 2)$ predicted contacts to show the $L$-dependent precision which is commonly used in both intra- and inter-protein contact predictions [24, 34, 42, 45–47]. However, it should be noted that if there are not $N$ or $L/K$ contacts in the native structures, the precisions will be underestimated when more than $N$ or $L/K$ contacts are considered. The other two are the accuracy order and the accuracy rate used by Zhao and Gong [35] to evaluate the performance of inter-chain contact predictions. The accuracy order is defined as the rank of first correct prediction divided by the total number of the residue pairs in the target. The accuracy rate is defined as the percentage of the targets with at least one successfully predicted contact when a certain number of predicted contacts are considered, compared to all the targets in the test set. Such accuracy order and accuracy rate parameters are useful if users want to validate the predicted contacts using experimental methods, because they will know how many pairs of residues need to be examined to get a positive result.

For docking applications, the quality of a predicted binding mode is measured by the TM-score [61, 62, 75] and the CAPRI criteria. If more than one top binding modes were considered for a target, the one with the highest TM-score was used to assess the docking performance for the target. For the CAPRI criteria, the quality is divided into four categories: high, medium, acceptable and incorrect [76]. The success rate is used to measure the docking performance, which is defined as the percentage of the targets with at least one successful (acceptable or better accuracy) prediction among the total number of targets in the test set when a certain number of top predictions are considered.

## Results and Discussion

### Overall Performance of DeepHomo

#### Evaluation on experimental PDB structures

**Fig. 2:**
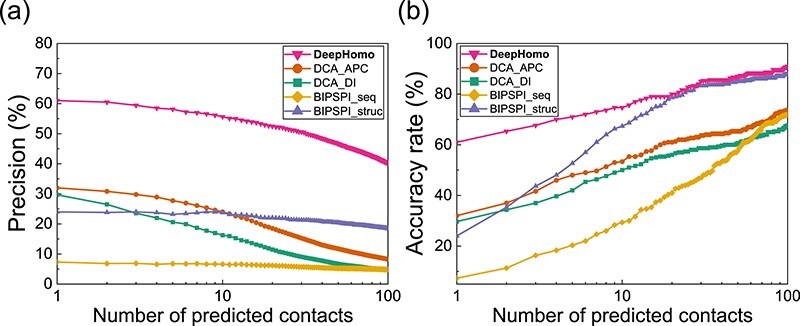
The performance of DeepHomo, two DCA-based approaches (DCA_DI and DCA_APC) and two machine learning-based methods (BIPSPI_seq and BIPSPI_struc) on the PDB test set of 300 experimental homo-dimeric structures. (A) The precision, number of TP predictions divided by number of predictions, as a function of the number of predicted contacts. (B) The accuracy rate, number of the targets with at least one successfully predicted contact divided by the total number of targets in a test set, as a function of the number of predicted contacts.

We first evaluated the performance of our DeepHomo model in inter-protein residue–residue contact prediction on a large test set of 300 experimental homo-dimeric complexes from the PDB. Figure 2 shows the precision and accuracy rate of DeepHomo in contact prediction as a function of the number of predicted contacts on the 300 homo-dimers. For comparison, the figure also shows the corresponding results of DCA-based methods and ML-based approaches on this test set. The precisions and accuracy rates for top 1, 10 and 100 predicted contacts are listed in Table 1. The top $L/K$ precisions and the accuracy orders are listed in Table 2. The DCA-based methods used as baselines in this study are similar to the Uguzzoni *et al*’s method [39]. First, based on the produced MSA, the direct coupling scores containing the raw direct information (DI) score and APC-corrected score were calculated using CCMpred. Then, according to the input monomer structure, the residue pairs with intra-monomer distances of more than 12 Å were regarded as the candidates of inter-protein contacts in the homo-oligomeric interface. At last, the candidate contacts were sorted by their co-evolutional scores, DI scores or APC scores, which represent DCA_DI and DCA_APC contact prediction approaches. BIPSPI is a ML method for the prediction of residue–residue contacts in the hetero-dimer interfaces, which employs extreme gradient boosting (XGBoost) models as a classifier [38]. It was trained on the Protein–Protein Docking Benchmark version 5.0 [77] and has achieved a good performance. BIPSPI has two versions that accepts the sequence as input (BIPSPI_seq) or the structure as input (BIPSPI_struc), respectively. We have tested both the sequential and structural versions by submitting jobs to the BIPSPI web server. The sequences and monomer structures uploaded to the web server were the same as those used by our DeepHomo model. After the jobs was done, the predicted results were downloaded from the BIPSPI web server and analyzed.

**Table 1:** Comparison of the precisions and accuracy rates by DeepHomo and the other four methods on the PDB test set of 300 experimental homo-dimeric complexes when the top 1, 10 and 100 predicted contacts are considered

		Precision (%)				Accuracy rate (%)	
Method	Top 1	Top 10	Top 100		Top 1	Top 10	Top 100
**DeepHomo**	**61.0**	**55.6**	**40.2**		**61.0**	**74.7**	**91.0**
DCA_APC	32.0	24.0	8.2		32.0	53.3	73.7
DCA_DI	29.7	16.3	4.8		29.7	50.0	67.7
BIPSPI_seq	7.3	6.6	4.9		7.3	29.3	72.0
BIPSPI_struc	24.0	23.8	18.4		24.0	67.3	87.7

**Table 2:** Comparison of the top $L/K (K=30, 20, 10, 5, 2)$ precisions and the accuracy orders by DeepHomo and the other four methods on the PDB test set of 300 experimental homo-dimeric complexes

			Precision (%)				Accuracy order(‰)
Method	Top L/30	Top L/20	Top L/10	Top L/5	Top L/2	
**DeepHomo**	**57.2**	**54.9**	**52.1**	**47.8**	**39.4**		**1.2**
DCA_APC	25.9	23.4	18.9	13.5	8.5		10.3
DCA_DI	18.2	15.7	11.9	7.8	4.9		25.4
BIPSPI_seq	7.0	6.7	6.0	5.6	4.8		5.8
BIPSPI_struc	23.3	23.2	22.5	20.9	18.6		3.8

It can be seen from Tables 1– 2 and Figure 2 that our DeepHomo model achieved a much better performance than DCA-based methods and ML-based methods. For the top 1, 10 and 100 predicted contacts, DeepHomo obtained the precisions of 61.0%, 55.6% and 40.2%, respectively, compared with 29.7%, 16.3% and 4.8% for DCA_DI, 32.0%, 24.0% and 8.2% for DCA_APC, 7.3%, 6.6% and 4.9% for BIPSPI_seq, and 24.0%, 23.8% and 18.4% for BIPSPI_struc (Table 1). For all the top $L/K (K=30, 20, 10, 5, 2)$ precisions, DeepHomo also achieved the best performance among the five methods and obtained very high precisions (Table 2). Similar advantages of DeepHomo over DCA-based and ML-based approaches can also be observed in the accuracy rate and the accuracy order of contact prediction. For example, DeepHomo gave a high accuracy rate of 91.0% for top 100 predicted contacts, which is significantly higher than 73.7% for DCA_APC, 67.7% for DCA_DI , 72.0% for BIPSPI_seq and 87.7% for BIPSPI_struc (Table 1). DeepHomo also obtained the best accuracy order of 1.2‰ among the five methods, which means that only the top 1.2‰ residue pairs need to be experimentally examined to find a true contact when all the residue pairs are ranked by our DeepHomo.

Although our deep learning model used the same MSA and monomer structure with DCA-based methods, the precision of DeepHomo almost doubled the precision of DCA_APC when the top predicted contact was considered. For top 100 predicted contacts, the improvement was much more significant and the precision has been improved almost over four times, compared with DCA_APC method. For DCA-based methods, DCA_APC method performed better than DCA_DI method for all the top 100 predicted contacts. This is consistent with the previous study in monomer protein contact prediction that APC helps improve the prediction quality [19, 78].

#### Application to realistic CASP-CAPRI targets

**Fig. 3:**
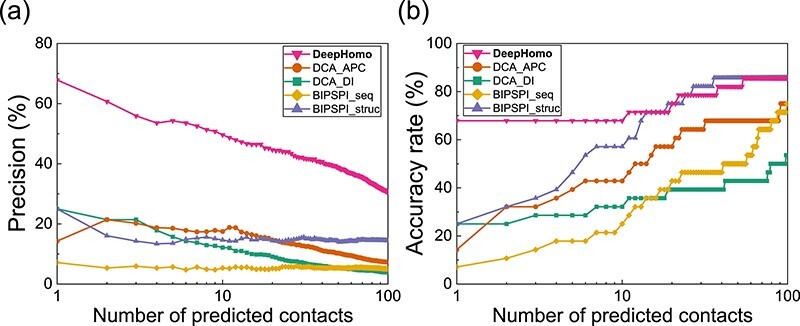
The performance of DeepHomo, two DCA-based approaches (DCA_DI and DCA_APC) and two machine learning-based methods (BIPSPI_seq and BIPSPI_struc) on the CASP-CAPRI of 28 realistic homo-dimeric targets. (A) The precision as a function of the number of predicted contacts. (B) The accuracy rate as a function of the number of predicted contacts.

As the monomer structure for a sequence is normally unknown in real applications, we have further tested our DeepHomo model on the CASP-CAPRI set of 28 realistic homo-oligomeric targets. To be consistent with the blind prediction challenge in CASP, we have used the top Zhang-Server model, which has been blindly predicted by the I-TASSRER server from the Zhang group [53, 54], as the monomer structure for the input of DeepHomo during the evaluation. Figure 3 shows the precision and accuracy rate of DeepHomo in residue contact prediction as a function of the number of predicted contacts on the CASP-CAPRI test set. For comparison, the figure also gives the corresponding results of other DCA-based methods and ML-based approaches. The precisions and accuracy rates for top 1, 10 and 100 contacts are listed in Table 3. The top $L/K$ precisions and the accuracy orders are listed in Table 4.

**Table 3:** Comparison of the precisions and the accuracy rates by DeepHomo and other four approaches on the CASP-CAPRI set of 28 realistic targets when the top 1, 10 and 100 predicted contacts are considered

		Precision (%)				Accuracy rate (%)	
Method	Top 1	Top 10	Top 100		Top 1	Top 10	Top 100
**DeepHomo**	**67.9**	**49.6**	**30.7**		**67.9**	**67.9**	**85.7**
DCA_APC	14.3	17.5	7.3		14.3	42.9	75.0
DCA_DI	25.0	12.1	3.9		25.0	32.1	53.6
BIPSPI_seq	7.1	5.4	5.1		7.1	25.0	75.0
BIPSPI_struc	25.0	14.6	14.5		25.0	57.1	85.7

**Table 4:** Comparison of the top $L/K (K=30, 20, 10, 5, 2)$ precisions and the accuracy orders by DeepHomo and the other four methods on the CASP-CAPRI set of 28 realistic targets

			Precision (%)				Accuracy order (‰)
Method	Top L/30	Top L/20	Top L/10	Top L/5	Top L/2		
**DeepHomo**	**51.2**	**47.1**	**43.2**	**37.4**	**28.2**		**2.1**
DCA_APC	17.9	16.8	12.4	9.4	6.0		22.6
DCA_DI	13.9	11.7	7.1	5.0	3.1		35.4
BIPSPI_seq	6.4	5.9	6.6	5.5	5.4		3.1
BIPSPI_struc	16.3	15.4	15.8	14.4	13.3		6.9

**Fig. 4:**
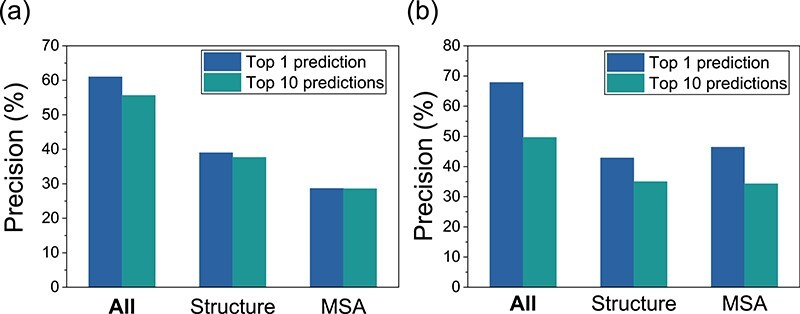
Ablation experiments of DeepHomo on the PDB test set of 300 experimental homo-dimeric structures (A) and the CASP-CAPRI test set of 28 realistic homo-dimeric targets (B). ‘All’ stands for the original DeepHomo model using all the features. ‘Structure’ stands for the model using only the features obtained from monomer structures. ‘MSA’ stands for the model using only the features obtained from MSAs.

It can be seen from Figure 3 that DeepHomo achieved the best performance among the five methods and gave a much higher precision and accuracy rate than the other four methods. Specifically, DeepHomo obtained the precisions of 67.9%, 49.6% and 30.7% for top 1, 10 and 100 predicted contacts, while the second-best method BIPSPI_struc gave much lower precisions of 25.0%, 14.6% and 14.5%, respectively (Table 3). DeepHomo also outperformed the other methods and gave very high top $L/K$ precisions (Table 4). Similar trends can also be observed in the accuracy rates and accuracy orders of different approaches, showing that DeepHomo yielded an overall much better performance than the other four approaches. Another notable feature in Figure 3B is that among the four approaches except DeepHomo, BIPSPI_struc obtained an overall better performance than the rest three approaches. Given the importance of structural features in contact prediction, this finding can be understood because BIPSPI_struc used the monomer structure as input in its contact prediction, while the other three approaches only took the sequence as input. Comparing Figures 2A and 3A, one can also find that DeepHomo maintained a comparably high precision of >60% on both the PDB test set of 300 homo-dimers and the CASP-CAPRI test set of 28 realistic targets. Given that the PDB test set consists of experimental monomer structures while the CASP-CAPRI test set is formed by blindly predicted models, the similar accuracy of DeepHomo on the two test sets suggested that DeepHomo is robust to the quality of monomer structures. This is especially valuable because the monomer structure is often unknown and needs to be predicted by a structure prediction method in realistic applications.

### Impact of MSA and monomer structure

#### Ablation studies of MSA and structural features

To investigate the effects of the MSA and the monomer structure on the performance of DeepHomo, we have conducted the ablation studies and tested the retrained models on the PDB test set of 300 homo-dimers and the CASP-CAPRI test set of 28 realistic targets. In the ablations studies, all the hyper-parameters were fixed, only the input features were changed during each experiment. The structural features include distance map, docking map, secondary structure and two hydrophobic features, and the MSA features consist of DCA scores and PSSM matrix. As shown in Figure 4, the structural features and MSA features are both very important to our model. With only the structural or MSA features as input, the precisions of top 1 and 10 predictions both decrease a lot on the two test sets. It is obviously that the MSA features is critical to our deep learning model where the information of spatial adjacencies between residues may be hidden in the evolutionary information. Compared with the MSA features, the structural features have a higher impact on the accuracy of DeepHomo on the PDB test set. However, on the CASP-CAPRI test set, the MSA features may be slightly more important than the structural features. This can be understood because the crystal structures of monomers were used for the PDB test set while the predicted monomer structures were used for the realistic CASP-CAPRI test set.

**Fig. 5:**
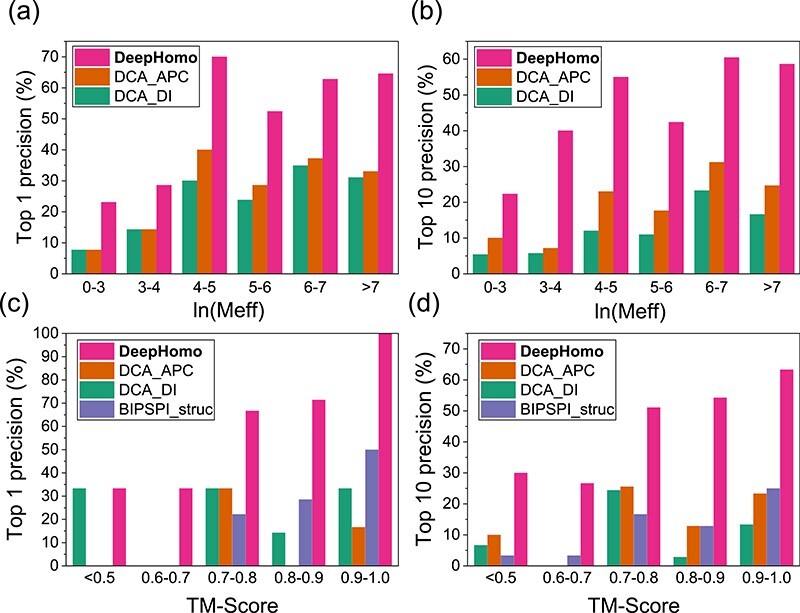
The impact of MSA depth and monomer structural quality. The precisions of top 1 (a) and top 10 (b) predicted contacts by DeepHomo, DCA_DI and DCA_APC with respect to the MSA depth measured by $\ln (M_{\textrm{eff}})$ on the PDB test set of 300 homo-dimeric structures. The precisions of the top 1 (c) and top 10 (d) predicted contacts by DeepHomo and other four methods with respect to the quality of monomer structures measured by TM-score on the CASP-CAPRI test set of 28 realistic targets.

**Fig. 6:**
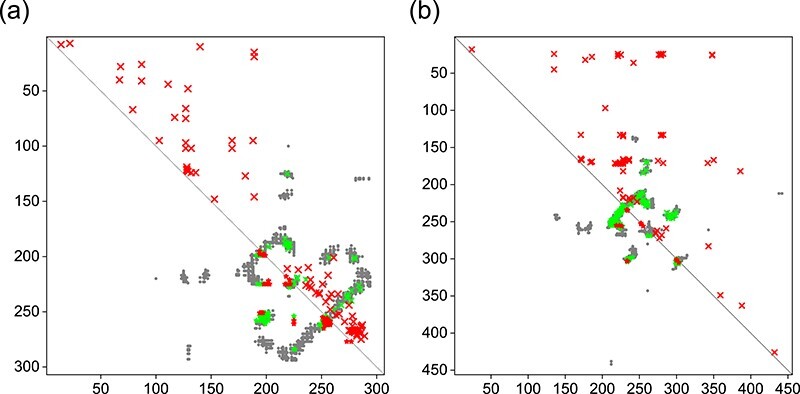
Comparison between the native contacts and the top 100 contacts predicted by DeepHomo (lower left triangle) and DCA_APC (upper right triangle) for T85 (A) and T93 (B). The native contacts, correct predictions and incorrect predictions are colored in gray, green and red, respectively. The diagonal line is colored in black.

#### Impact of the MSA depth

As the MSA is crucial to our DeepHomo model and other DCA-based approaches, we have examined the effect of the MSA depth on the performance of our method. Here, the effective number of sequence homologs in an MSA, $M_{\textrm{eff}}$, was used to measure the MSA depth. $M_{\textrm{eff}}$ can be regarded as the number of non-redundant sequence homologs in an MSA under a sequence identity cutoff. The sequence identity between two sequences in an aligned MSA is defined as the percentage of the positions with corresponding residues. In this study, a sequence identity cutoff of 70% was used to remove redundancy. The $M_{\textrm{eff}}$ is calculated as follows:

(1) \begin{align*}& M_{\textrm{eff}}=\sum_{i=1}^{M} 1 / m_{i} \end{align*}

where $M$ is the number of sequences in an MSA and $m_{i}$ is the number of sequences that have sequence identity >70% with the $i$-th sequence in the MSA.

Figures 5A and 5B show that the precisions of top 1 and top 10 predicted contacts with respect to $\ln (M_{\textrm{eff}})$ on the test set. The first three intervals were merged together because of the small number of proteins in these intervals. It can be seen from the figure that our DeepHomo method outperformed the two DCA-based approaches regardless of the number of $M_{\textrm{eff}}$. As the evolutionary information is included in the MSA, all methods generally obtained a better performance with increased $M_{\textrm{eff}}$ for both top 1 and top 10 predicted contacts. A higher $M_{\textrm{eff}}$ generally leads to better coevolutionary scores for the DCA-based methods and evolutionary input features for our deep learning model, which resulted in a better performance of all tested methods. It is interesting to note that the top 1 and 10 precisions in the 4–5 range show unexpected high values. This may be attributed to the much larger interfaces of the targets in this range. The average interface area for the targets in the 4–5 range is 2240 Å$^{2}$, compared with 1797 Å$^{2}$ for the 5–6 range, 1933 Å$^{2}$ for the 6–7 range and 2026 Å$^{2}$ for the >7 range, respectively. Target with larger interface may have more inter-protein contacts, which may make it easier to predict the contacts. In addition, despite the high accuracy for the cases of $\ln (M_{\textrm{eff}})>6$ (approximately $M_{\textrm{eff}}>400$), our DeepHomo model also obtained good accuracies in the 4–5 and 5–6 ranges of $\ln (M_{\textrm{eff}})$ and gave the precisions of 70.0% and 52.4% for top 1 predicted contact and 55.0% and 42.4% for top 10 predicted contacts, respectively. This suggests that DeepHomo is able to learn correct contact information from not very deep MSAs with the help of other information such as structural features.

#### Impact of monomer structural quality

To investigate the impact of the quality of monomer structures, we have examined the performance of our DeepHomo model on the monomer structures with different accuracies in the CASP-CAPRI test set. Here, TM-score [61] was used to measure the quality of a monomer structure. For each target in the CASP-CAPRI test set, the TM-score between the predicted monomer structure and the native structure was calculated using TM-align [60]. Figure 5C and 5D give the average precisions of DeepHomo for top 1 and top 10 predicted contacts in the different bins of TM-score. For comparison, the figure also shows the corresponding results of other three approaches, DCA_DI, DCA_APC and BIPSPI_struc. Here, BIPSPI_seq was excluded because it does not need monomer structures in the contact prediction. In addition, as no TM-score is in the range 0.5-0.6, no result is shown for this interval in the figure. All targets with TM-score<0.5 were merged in one interval [62].

From Figures 5C and 5D, one can see a general trend that DeepHomo and BIPSPI_struc show better accuracies for better-quality monomer structures with higher TM-scores, while the performance trend is not very clear for the DCA-based methods. It can also be seen from Figures 5C and 5D that the TM-score of 0.7 seems to be a threshold for different approaches. On the one hand, the accuracies of the DCA-based methods for TM-score>0.7 become much better than those for TM-score <0.7 when the top 10 predicted contacts were considered. This may be understood because the targets with TM-score <0.7 tend to be hard targets and do not have many homologous sequences. Therefore, the DCA-based approaches would not be able to extract significant coupling information due to the limited number of sequences in the MSA. On the other hand, DeepHomo performed better with the increased quality of monomer structures for TM-score>0.7, and reached a stable precision of about 32% for TM-score<0.7 when the top predicted contact was considered. From Figure 5C and 5D, one can also see that overall DeepHomo and BIPSPI tend to depend on the TM-score of monomer structures more than the DCA-based approaches. This can be understood because both DeepHomo and BIPSPI take structural features extracted from the monomer structure as input. The tendency of the precisions for the first two bins (<0.5 and 0.6-0.7) is slightly different. The top 1 precision does not change, while the top 10 precision decreases slightly with the increasing TM-score, which may be attributed to the insufficient statistics becasue there are only three targets for each bin. However, for the DCA-based methods, the monomer structure was only used as a filter in the post process, so these methods rely less on the quality of the monomer structures. Despite the impact of structural quality, our DeepHomo model can achieve a good precision of 66.7% for targets with TM-score in 0.7-0.8 for the top predicted contact. This means that our deep learning model is accurate enough to learn correct contact information with only moderate-quality monomer structures.

In addition to assessing our DeepHomo using the monomer models predicted by the Zhang-Server on the CASP-CAPRI test set, We have also evaluated our DeepHomo model using the native monomer structures, whose results are shown in Supplementary Tables S1 and S2. As there were some missing residues in the PDB structures, the predicted monomer models were first trimmed according the length of the native structures to ensure a fair comparison. Then, the trimmed monomer models and the native structures were used to produce different structural features, where the sequential features were the same, which were obtained from the same MSAs. It can be seen from Supplementary Tables S1 and S2 that using the true native monomer structures for the prediction of inter-protein contacts achieved a better performance than using the predicted monomer structures. Specifically, DeepHomo obtained a precision improvement of around 5% when using the native structures. Improvement of the accuracy rate and accuracy order can also be observed in Supplementary Tables S1 and S2. These results again demonstrated the importance of the quality of monomer structures for the inter-protein contact prediction.

**Fig. 7:**
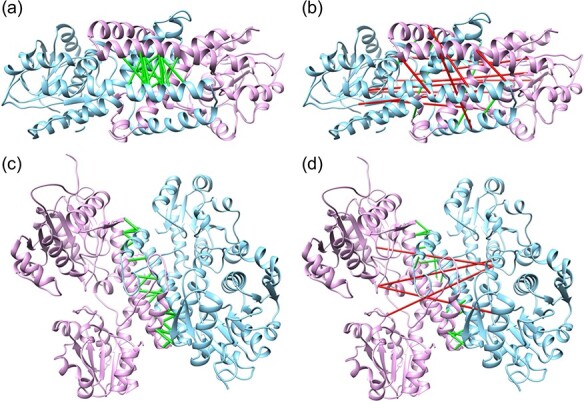
Top 10 predicted contacts by DeepHomo (A and C) and DCA_APC (B and D) on T85 (A and B) and T93 (C and D). The two monomers of native homo-dimeric structures are shown in ribbons and colored in pink and blue, respectively. The correct and wrong predictions are shown in green and red connections.

**Fig. 8:**
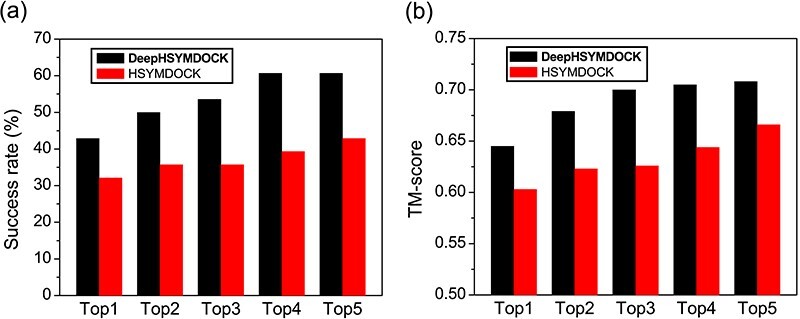
Success rates and TM-scores of DeepHSYMDOCK, i.e. DeepHomo+HSYMDOCK, and *ab initio* HSYMDOCK docking algorithms in binding mode prediction on the CASP-CAPRI test set of 28 realistic targets when top one to five predictions were considered.

### Examples of contact prediction

Figure 6 shows two selected examples of the top 100 predicted contacts by our DeepHomo model and the DCA_APC method versus the native contact map for targets T85 and T93 in the CASP-CAPRI test set. Comparisons between DeepHomo and the other three methods (DCA_DI, BIPSPI_seq and BIPSPI_struc) are shown in Supplementary Figure S4A. It can be seen from the figures that the predicted contacts by our DeepHomo model are all distributed near the native ones and can grab the important interaction mode. However, the predicted contacts by the DCA-based methods are very dispersed across all the contact map, which results in many false positive predicted contacts, while the predictions by BIPSPI methods are more concentrated but with many false positive predictions. Specifically, DeepHomo achieved the high precisions of 56% and 87% on the two targets of T85 and T93, while the precisions of the DCA_APC method were only 25% and 31% with 0% and 28% for DCA_DI, 19% and 3% for BIPSPI_seq and 17% and 29% for BIPSPI_struc, respectively. From Figure 6, one can also see that the predicted contacts by DeepHomo are mostly close to the native contacts on the contact map, even though they may not overlap. That means that such near-native contacts by DeepHomo are still roughly correct, even though they may be classified to be incorrect contacts according the cutoff of 8 Å. In contrast, for the DCA_APC method, many of its predicted contacts are far from the native ones and thus are truly wrong contacts (Figure 6). Figure 7 shows a comparison of the top 10 predicted contacts by DeepHomo and DCA_APC in the native homo-dimeric structures of T85 and T93. The top 10 predictions by other three methods are shown in Supplementary Figure S4B. It can be seen from the figure that our DeepHomo model successfully predicted all the contacts and achieved an precision of 100% on targets T85 and T93 for top 10 predicted contacts, while the DCA_APC method only gave correct predictions for 40% and 80% of the native contacts on these two targets with 0% and 90% for DCA_DI, 40% and 0% for BIPSPI_seq and 10% and 20% for BIPSPI_struc, respectively (Figure 7 and Supplementary Figure S4). These results again suggest the accuracy and robustness of our DeepHomo approach.

### Integration of DeepHomo into protein docking

Accurate prediction of inter-protein contacts will greatly help the structure determination of corresponding protein–protein complexes. To investigate the practical role of DeepHomo in complex structure prediction, we have integrated the predicted contacts by DeepHomo into our *ab initio* HSYMDOCK symmetric docking program by applying the predicted contacts in a post-docking filter, named as DeepHSYMDOCK, and evaluated its performance on the CASP-CAPRI test set of 28 realistic targets. For each target, the same monomer structure, i.e. the first Zhang-Server model, was used as the input structure for the DeepHomo and HSYMDOCK processes of DeepHSYMDOCK. All the default parameters were used during the HSYMDOCK docking calculations. Given that the accuracy of contact prediction is critical to the docking process, only the top predicted contact by DeepHomo was used to filter the final binding modes predicted by HSYMDOCK.

Figure 8 shows the success rates of DeepHSYMDOCK and HSYMDOCK in binding mode prediction on the CASP-CAPRI test set. It can be seen from the figure that the predicted contacts by DeepHomo did significantly improve the docking performance in complex structure prediction, When the top five predictions are considered, DeepHSYMDOCK achieved a success rate of 42.9% and 60.7% for top 1 and 5 predictions, which is considerably higher than 32.1% and 42.9% for the *ab initio* HSYMDOCK docking program. Similar trends can also be observed in the TM-scores of docked complex structures, where the TM-score was calculated using MMalign [75]. Namely, DeepHSYMDOCK obtained an average TM-score of 0.645 and 0.708 for the top 1 and 5 predictions, respectively, compared to 0.603 and 0.666 for HSYMDOCK. Without using contacts, HSYMDOCK did not predicted any correct complex structures within the top five predictions for T85 and T93. However, with the help of the top predicted contacts, DeepHSYMDOCK achieved a medium-accuracy binding mode with $L_{\textrm{rmsd}}$=2.804 Å, $I_{\textrm{rmsd}}$=2.247 Å, $f_{\textrm{nat}}$=60.211% and TM-score$=0.942$ for T85, and an acceptable-accuracy binding mode with $L_{\textrm{rmsd}}$=7.441 Å, $I_{\textrm{rmsd}}$=3.446 Å, $f_{\textrm{nat}}$=29.167% and TM-score$=0.816$ for T93, within the top predictions. These results demonstrated the important role of DeepHomo in the structure prediction of protein–protein complexes.

## Conclusions

We have presented a deep learning model for inter-protein residue–residue contact prediction across homo-oligomeric protein interfaces by integrating both structural and MSA features of monomers, named as DeepHomo. Our DeepHomo model was extensively evaluated on two independent data sets, the PDB test set of 300 experimental structures and the CASP-CAPRI test set of 28 realistic homo-dimeric targets, and compared with state-of-the-art DCA-based methods (DCA_APC and DCA_DI) and ML-based approaches (BIPSPI_seq and BIPSPI_struc). It was shown that DeepHomo achieved a high accuracy in inter-protein contact prediction and outperformed existing DCA and ML-based methods. DeepHomo was robust to the depth of MSAs and the quality of monomer structures, and can achieve good accuracies for the MSA of $\sim $100 sequences and the monomer structure of TM-score$\sim $0.7. Integrating the predicted contacts into protein–protein docking significantly improved the docking accuracy, suggesting the practical role of DeepHomo in the determination of homo-dimeric structures. The present model demonstrated the efficacy of integrating both structural and sequential features into a deep learning model for accurate inter-protein residue–residue contact predictions.

**Key Points**

- Protein–protein contacts in homo-oligomeric complexes were accurately predicted using a deep learning model.
- Unlike traditional approaches, our model includes distance and docking maps based on monomer structures.
- Our model much outperformed existing direct-coupling analysis and machine learning-based approaches on realistic complexes.
- Our model was robust to monomer structures and still performed well with predicted protein structures like CASP models.
- Integrating predicted contacts into protein–protein docking significantly improved the docking accuracy.

## Availability

DeepHomo is available at http://huanglab.phys.hust.edu.cn/DeepHomo/. The package consists of the processed files of the datasets, the trained model, source code and a step-by-step instruction for users.

## Supplementary Material

Suppl_bbab038

## Notes

**Yumeng Yan** is a PhD student in the School of Physics at the Huazhong University of Science and Technology. His research interests include molecular docking and protein–protein contact prediction.

**Sheng-You Huang** is a full professor in the School of Physics at the Huazhong University of Science and Technology. His research interests are molecular docking and scoring functions for protein–ligand interactions, protein–protein interactions and protein–peptide interactions. More information about his research group can be found at http://huanglab.phys.hust.edu.cn.
