# Supplementary material for: Accurate prediction of inter-protein residue–residue contacts for homo-oligomeric protein complexes
Source: Brief Bioinform. 2021 Mar 5;22(5):bbab038. doi: 10.1093/bib/bbab038 (PMC8425427; doi:10.1093/bib/bbab038)
Supplement: Suppl_bbab038 [file suppl_bbab038.pdf]

Supplementary Material:

Accurate prediction of inter-protein residue-residue contacts for homo-oligomeric protein complexes

Yumeng Yan and Sheng-You Huang*

School of Physics, Huazhong University of Science and Technology, Wuhan, Hubei 430074, P. R. China

Key Words: protein-protein interaction, homo-oligomers, protein-protein docking, inter-protein contact prediction, deep learning

Running Title: Contact prediction for homo-oligomeric complexes

*Email: huangsy@hust.edu.cn; Phone: +86-27-87543881; Fax: +86-027-87556576

1 Overall Performance of DeepHomo with contact threshold of 6 Å

To investigate the effect of the distance threshold for inter-protein contacts on the performance, another threshold of 6 Å used by BIPSPI [1] was also adopted to define the residue-residue contact. The DeepHomo model was then re-trained with the same hyper-parameters and the network using the new labels. The new model was also evaluated on the PDB set with 300 experimental homo-dimeric complexes and the CAPRI-CASP set with 28 realistic targets. The two DCA-based methods and two machine learning-based methods were also re-evaluated for comparison. The results for the PDB set are listed in Tables S3 and S4 and the results for the CAPRI-CASP set are listed in Tables S5 and S6. Figure S1 shows the comparison between our new DeepHomo model and the other four methods in terms of the precision and accuracy rate. It can be seen the tables and figure that our new DeepHomo model still outperformed the other methods on the both test sets under almost all the metrics. For example, DeepHomo achieved the top L/2 precision of 25.9% on the PDB set while the second best method BIPSPI_struc obtained the precision of only 10.3%. It should be noted that all five methods have shown a decline in the performance when a smaller threshold was used. This can be understood because a smaller threshold reduces the number of defined true contacts and thus make the prediction more challenging.

2 Comparison between contact-based model and distance-based model

Recently, distance-based folding has shown its great power in the monomer structure prediction [2–4]. Thus, we have also tried to use the similar strategy to train a distance-based deep learning model for the prediction of distance distribution of inter-protein residue pairs for homo-oligomers. The minimal distance of heavy atoms between two residues was discretized into 15 bins: $<$4.0 Å, 4.0 to 6.0 Å, 6.0 to 8.0 Å, $\cdots$, 28.0 to 30 Å, $>$30Å. Then, the prediction of the distance distribution was translated into multi-class classification. The architecture of the neural network was almost the same as that for the contact-based model except that the activation function of the output layer was changed from the sigmiod to softmax. The distance-based model was also tested on the PDB set and the CASP-CAPRI set. Figure S2 shows the precision as a function of the number of predicted contacts when using

different contact thresholds (6 Å and 8 Å). For comparison, the results of the contact-based models trained with respective contact thresholds are also shown. When using the threshold of 6 Å, the contact probability of a residue pair predicted by the distance-based model is the sum of the predicted probability of the first two bins, and for threshold of 8 Å, the probability is the sum of the first three bins. As shown in Figure S2, the distance-based model does not outperform the contact-based models whether the distance threshold is 6 Å or 8 Å. As the residue pairs in contact are only a small part of all the possible residue pairs. When the distance is discretized into multi-bins, the residue pairs in each bin become even fewer, which will make the prediction more difficult. In addition, the conformational changes in the monomer structures would also lead to some fluctuations in the distance of inter-protein residues. More sophisticated distance-based model and network architectures may be needed to handle such challenging situations in the future.

3 Application to homo-oligomers with more than two chains

Except C2 symmetry, homo-oligomers may form other symmetry types like C3, D2 with more than two chains. We have also evaluated the performance of our DeepHomo model on the homo-oligomers consisting of more than two chains on our symmetric protein docking benchmark (SDB) [5], which is a comprehensive and non-redundant benchmark for symmetric protein docking and consists of targets with different types of symmetry. The targets with C2 symmetry were removed from the data set as we focused on the homo-oligomers with more than two chains. The targets with tetrahedral and helical symmetry were also removed because HSYMDOCK [6, 7] only supports Cn and Dn symmetry so far. The 69 retained targets consisting of C3-C7 symmetry and D2-D6 symmetry, which were used to evaluate the performance of our DeepHomo model and the other four methods. The input features of these targets were produced by the same process as that for C2 symmetry, except the docking map feature which was produced with restriction of the respective symmetry type of the target. The ground truth label for each residue pair was defined according to the minimal distance of the pair in the multiply interfaces. From the results shown in Tables S7-S8 and Figure S3, we can see that our DeepHomo model shows the best performance on the metrics of precision, accuracy rate and accuracy order when compared with the other four methods. Although our DeepHomo model was trained on

the data set with C2 symmetry, it also achieved good performance on the targets with other symmetry types. However, the performance decreases compared with that on the test set consisting of targets with only C2 symmetry. This can be understood as follows. When training the DeepHomo model, the docking map was produced with the constraint of C2 symmetry, so the information of C2 symmetry was already included in the DeepHomo model. As the oligomerization state can be predicted from the sequence [8,9], the information of C2 symmetry may also be implicit in the sequential features and in the trained model. Therefore, DeepHomo is expected to perform better on C2 targets than others. As there are not enough data for homo-oligomers with other symmetry types, transfer learning [10, 11] might be used in the future to improve the performance of our model on homo-oligomers with more than two chains.

References

(1) Sanchez-Garcia R, Sorzano COS, Carazo JM, Segura J. BIPSPI: a method for the prediction of partner-specific protein-protein interfaces. Bioinformatics. 2019;35(3):470-477.

(2) Yang J, Anishchenko I, Park H, Peng Z, Ovchinnikov S, Baker D. Improved protein structure prediction using predicted interresidue orientations. Proc Natl Acad Sci U S A. 2020;117(3):1496-1503.

(3) Xu J. Distance-based protein folding powered by deep learning. Proc Natl Acad Sci U S A. 2019;116(34):16856-16865.

(4) Senior AW, Evans R, Jumper J, et al. Improved protein structure prediction using potentials from deep learning. Nature. 2020;577(7792):706-710.

(5) Yan Y, Huang S-Y. A non-redundant benchmark for symmetric protein docking. Big Data Mining and Analytics. 2:92-99, 2019.

(6) Yan Y, Huang SY. CHDOCK: a hierarchical docking approach for modeling Cn symmetric homo-oligomeric complexes. Biophys. Rep. 2019;5(2):65-72.

(7) Yan Y, Tao H, Huang SY. HSYMDOCK: a docking web server for predicting the structure of protein homo-oligomers with Cn or Dn symmetry. Nucleic Acids Res. 2018;46(W1):W423-W431.

(8) Simeon S, Shoombuatong W, Anuwongcharoen N. et al. osFP: a web server for predicting the oligomeric states of fluorescent proteins. J Cheminform. 2016;8:72.

(9) Naveed H, Jackups R, Liang J. Predicting weakly stable regions, oligomerization state, and protein-protein interfaces in transmembrane domains of outer membrane proteins. Proc Natl Acad Sci U S A. 2009;106(31):12735-12740.

(10) Singh J, Hanson J, Paliwal K, et al. RNA secondary structure prediction using an ensemble of two-dimensional deep neural networks and transfer learning. Nature communications. 2019;10(1):1-13.

(11) Wang S, Li Z, Yu Y, et al. Folding membrane proteins by deep transfer learning. Cell systems. 2017;5(3):202-211.e3.

Table S1: Comparison of the precisions and the accuracy rates by DeepHomo with the structural features obtained from native monomer structures and Zhang-Server predicted models when the top 1, 10 and 100 predicted contacts are considered.

	Precision (%)			Accuracy rate (%)		
Method	Top 1	Top 10	Top 100	Top 1	Top 10	Top 100
**DeepHomo_Native**	**64.3**	**52.5**	**33.9**	**64.3**	**78.6**	**85.7**
DeepHomo_Zhang	60.7	46.4	29.9	60.7	67.9	85.7

Table S2: Comparison of the top $L/K(K = 30, 20, 10, 5, 2)$ precisions and the accuracy order by DeepHomo with the structural features obtained from native monomer structures and Zhang-Server predicted structures on the CASP-CAPRI set of 28 realistic targets.

	Precision (%)					Accuracy order (‰)
Method	Top L/30	Top L/20	Top L/10	Top L/5	Top L/2	
**DeepHomo_Native**	**51.1**	**49.4**	**45.7**	**38.9**	**31.7**	**2.0**
DeepHomo_Zhang	47.5	44.7	40.8	34.1	28.2	2.6

Table S3: Comparison of the precisions and accuracy rates by DeepHomo and the other four methods on the PDB test set of 300 experimental homo-dimeric complexes when the top 1, 10 and 100 predicted contacts are considered with the distance threshold of 6 Å.

	Precision (%)			Accuracy rate (%)		
Method	Top 1	Top 10	Top 100	Top 1	Top 10	Top 100
**DeepHomo**	**57.7**	**47.9**	**26.1**	**57.7**	**77.7**	**92.3**
DCA_APC	27.7	20.2	5.6	27.7	48.7	66.0
DCA_DI	26.7	12.9	3.0	26.7	44.3	59.3
BIPSPI_seq	4.7	3.0	2.2	4.7	19.7	56.0
BIPSPI_struc	18.0	14.8	10.4	18.0	58.0	86.0

Table S4: Comparison of the top $L/K(K = 30, 20, 10, 5, 2)$ precisions and the accuracy order by DeepHomo and the other four methods on the PDB test set of 300 experimental homo-dimeric complexes with the distance threshold of 6 Å.

	Precision (%)					Accuracy order (‰)
Method	Top L/30	Top L/20	Top L/10	Top L/5	Top L/2	
**DeepHomo**	**49.9**	**46.9**	**42.2**	**35.7**	**25.9**	**1.0**
DCA_APC	21.7	19.5	15.2	10.4	6.0	18.0
DCA_DI	15.1	12.6	9.1	5.8	3.3	35.9
BIPSPI_seq	3.6	3.4	2.9	2.8	2.3	9.8
BIPSPI_struc	15.2	14.8	13.7	12.3	10.3	4.6

Table S5: Comparison of the precisions and the accuracy rates by DeepHomo and other four approaches on the CASP-CAPRI set of 28 realistic targets when the top 1, 10 and 100 predicted contacts are considered with the distance threshold of 6 Å.

	Precision (%)			Accuracy rate (%)		
Method	Top 1	Top 10	Top 100	Top 1	Top 10	Top 100
**DeepHomo**	**57.1**	**44.6**	**20.9**	**57.1**	**75.0**	**82.1**
DCA_APC	14.3	15.0	5.1	14.3	39.3	64.3
DCA_DI	21.4	9.6	2.7	21.4	25.0	46.4
BIPSPI_seq	7.1	2.9	2.4	7.1	14.3	64.3
BIPSPI_struc	17.9	7.1	7.6	17.9	39.3	78.6

Table S6: Comparison of the top $L/K(K = 30, 20, 10, 5, 2)$ precisions and the accuracy order by DeepHomo and the other four methods on the CASP-CAPRI set of 28 realistic targets with the distance threshold of 6 Å.

	Precision (%)					Accuracy order (‰)
Method	Top L/30	Top L/20	Top L/10	Top L/5	Top L/2	
**DeepHomo**	**46.7**	**40.2**	**31.3**	**26.1**	**17.8**	6.1
DCA_APC	15.1	14.2	10.2	6.9	4.1	35.7
DCA_DI	10.4	8.6	5.2	3.2	1.9	68.2
BIPSPI_seq	3.4	2.5	3.0	2.5	2.7	**4.8**
BIPSPI_struc	9.9	9.1	8.4	7.3	7.2	10.0

Table S7: Comparison of the precisions and the accuracy rates by DeepHomo and the other four approaches on SDB set of 69 targets with more than two chains when the top 1, 10 and 100 predicted contacts are considered.

	Precision (%)			Accuracy rate (%)		
Method	Top 1	Top 10	Top 100	Top 1	Top 10	Top 100
**DeepHomo**	**33.3**	**27.4**	**19.3**	**33.3**	58.0	**85.5**
DCA_APC	18.8	8.8	4.2	18.8	33.3	72.5
DCA_DI	4.3	5.2	3.3	4.3	26.1	66.7
BIPSPI_seq	17.4	12.0	9.5	17.4	40.4	71.0
BIPSPI_struc	27.5	24.6	18.9	27.5	**60.9**	85.5

Table S8: Comparison of the top $L/K(K = 30, 20, 10, 5, 2)$ precisions and the accuracy order by DeepHomo and the other four methods on the SDB set of 69 targets with more than two chains.

	Precision (%)					Accuracy order (‰)
Method	Top L/30	Top L/20	Top L/10	Top L/5	Top L/2	
**DeepHomo**	**33.2**	**30.9**	**27.5**	**24.1**	**21.9**	**8.3**
DCA_APC	11.3	9.5	8.4	6.7	4.9	16.5
DCA_DI	6.4	5.6	5.3	4.4	3.3	63.9
BIPSPI_seq	15.3	14.8	12.8	12.1	10.0	15.3
BIPSPI_struc	23.8	22.8	24.0	22.7	20.3	10.8

Figure S1

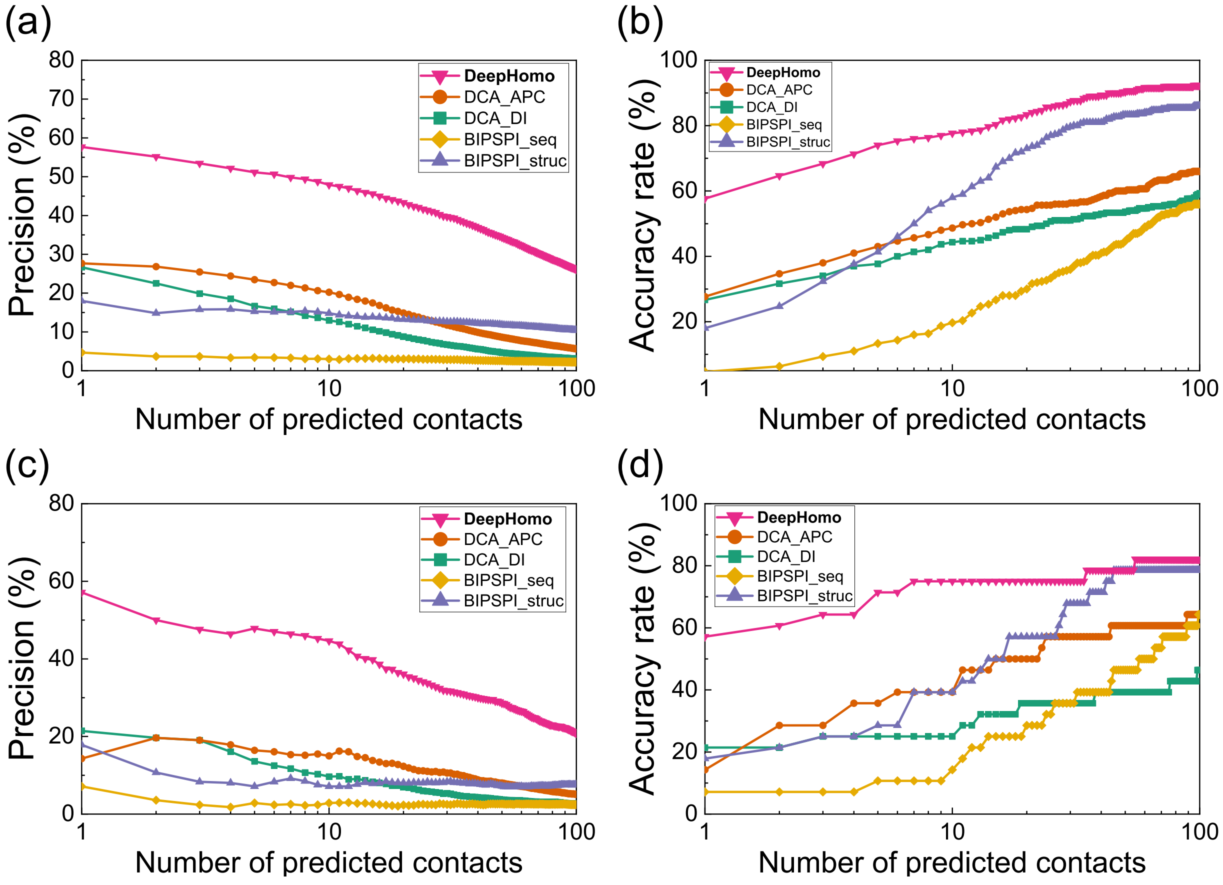

Figure S1: Performance of DeepHomo and the other four approaches with the threshold of 6 Å for contacts. The precision, number of true positive (TP) predictions divided by number of predictions, as a function of the number of predicted contacts on the PDB test set (a) and the CASP-CAPRI set (c). The accuracy rate, number of the targets with at least one successfully predicted contact divided by the total number of targets in a test set, as a function of the number of predicted contacts on the PDB test set (b) and the CASP-CAPRI set (d).

Figure S2

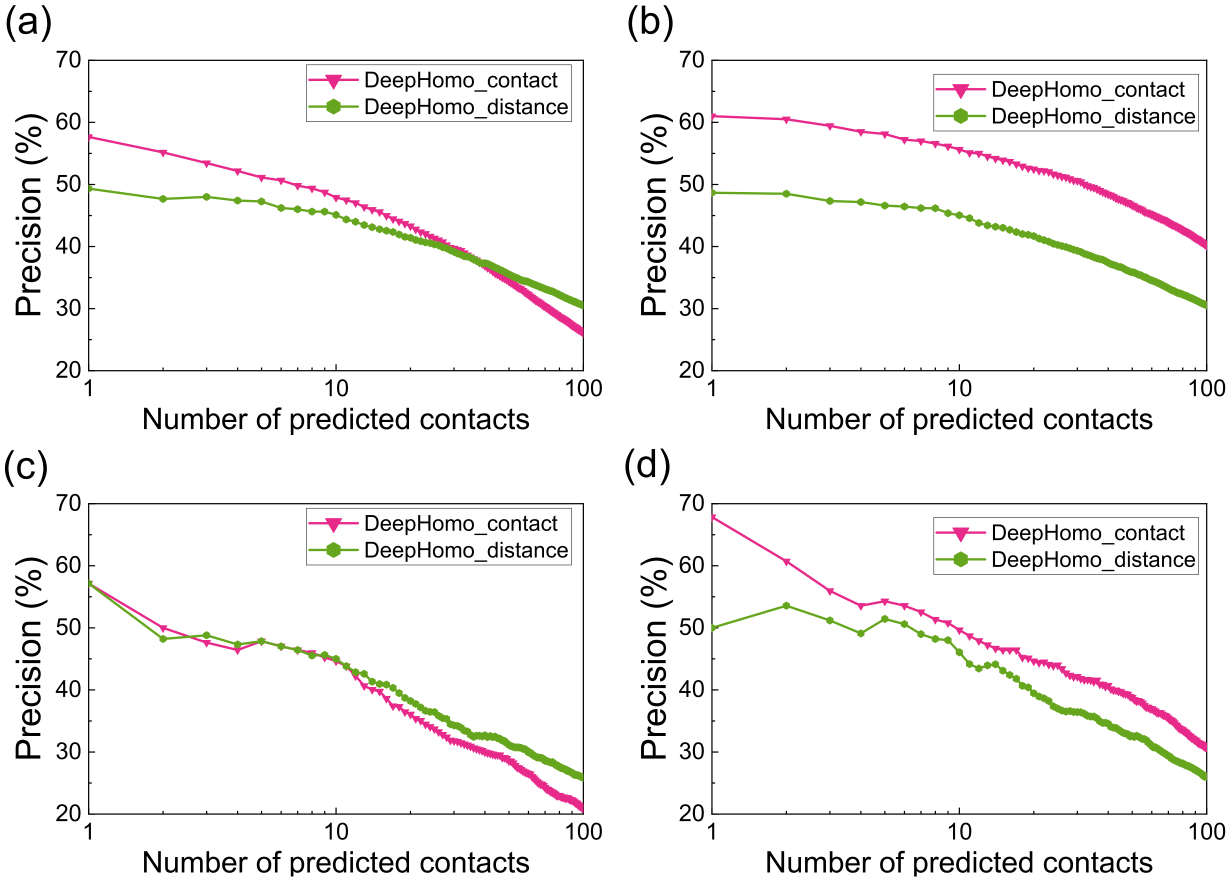

Figure S2: Performance of the contact-based model and the distance-based model on the PDB test set of 300 experimental homo-dimeric structures (a,b) and on the CASP-CAPRI test set of 28 realistic targets (c, d) with contact threshold of 6 Å (a,c) and 8 Å (b,d).

Figure S3

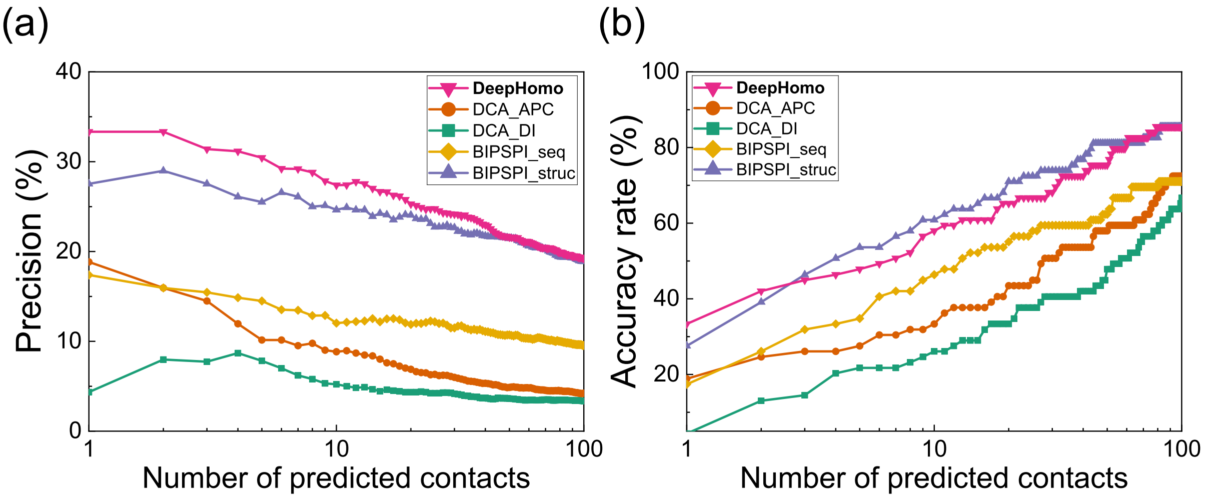

Figure S3: Performance of DeepHomo and the other four approaches on the SDB test set of 69 targets with more than two chains. (a) The precision, number of TP predictions divided by number of predictions, as a function of the number of predicted contacts. (b) The accuracy rate, number of the targets with at least one successfully predicted contact divided by the total number of targets in a test set, as a function of the number of predicted contacts.

Figure S4

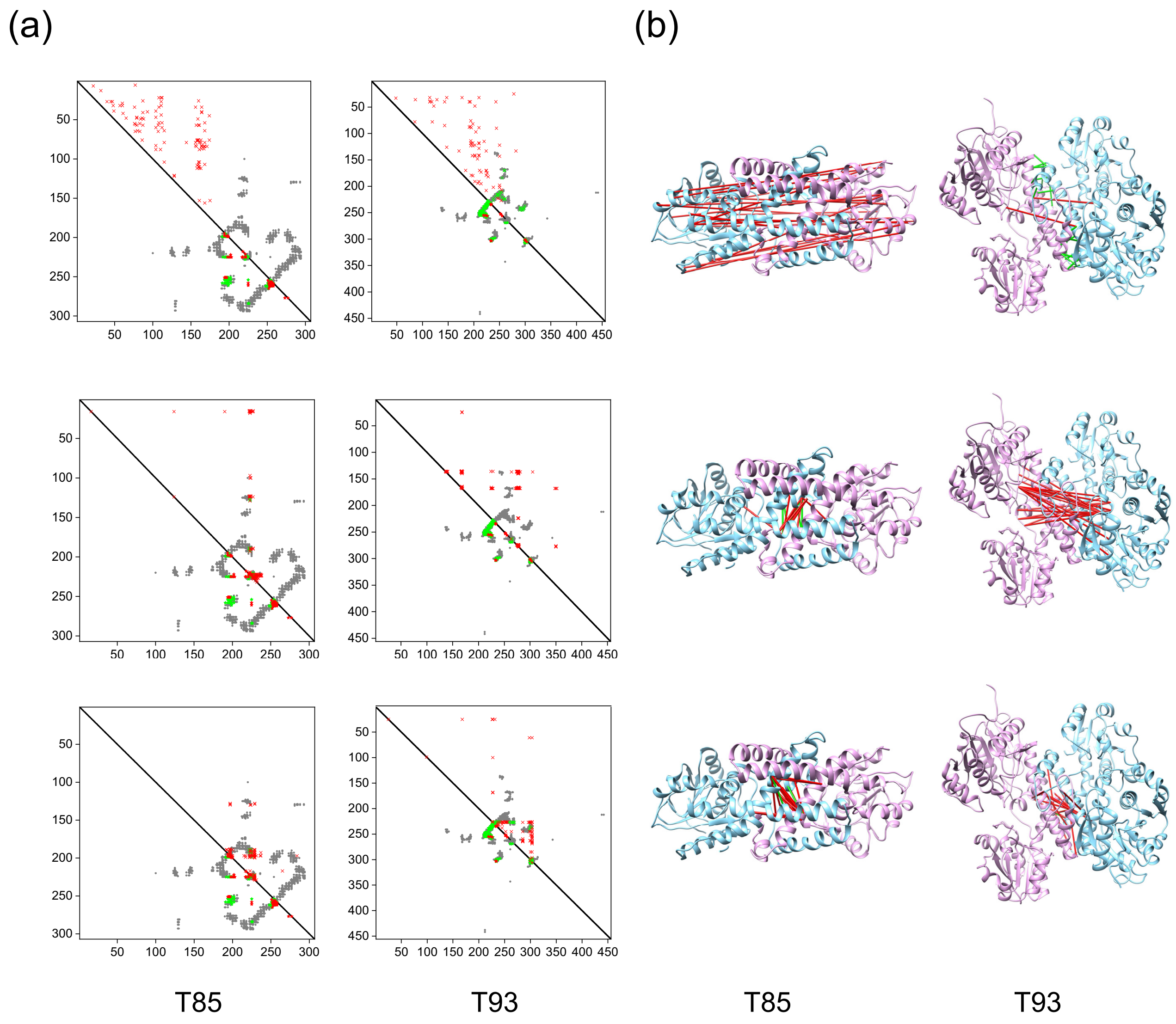

Figure S4: Comparisons between the native contacts and the top 100 contacts predicted by DeepHomo (lower left triangle) and other three methods (upper right triangle) (a) and the top 10 predicted contacts by the corresponding methods shown in 3D structures for T85 and T93 (b). From top to bottom are the performance comparisons and predicted contacts of DCA_DI, BIPSPI_seq, BIPSPI_struc, respectively. The same color schemes as Figure 6 and 7 are used.
